# Features of Kidney Function in Patients With Comorbidity of Arterial Hypertension and Chronic Obstructive Pulmonary Disease

**DOI:** 10.7759/cureus.31828

**Published:** 2022-11-23

**Authors:** Olha Boiko, Viktoriia Rodionova, Luydmyla Shevchenko

**Affiliations:** 1 Occupational Diseases, Clinical Immunology and Clinical Pharmacology, Dnipro State Medical University, Dnipro, UKR; 2 Department of Physical, Organic and Inorganic Chemistry, Oles Honchar Dnipro National University, Dnipro, UKR

**Keywords:** glomerular filtration rate, cystatin c, renal function, chronic obstructive pulmonary disease, hypertension

## Abstract

Background and objective

Chronic kidney disease (CKD) and hypertension are closely linked in terms of cause and effect. Decreased renal function is usually associated with increased blood pressure, and a steady increase in blood pressure accelerates the decline in renal function. In this study, we aimed to investigate laboratory parameters of renal function - blood creatinine level, urine creatinine level, urea blood level, urine albumin level, and in particular, serum cystatin C level - as early predictors of kidney damage and assess the filtration function of the kidneys based on the glomerular filtration rate (GFR) in patients with isolated arterial hypertension, those with a comorbid pathology of hypertension and chronic obstructive pulmonary disease (COPD). and those with isolated COPD.

Materials and methods

The study included a total of 101 patients (the final sample consisted of 88 patients) with hypertension and COPD, who were divided into three groups: Group I consisted of 38 patients with hypertension, Group II comprised 27 patients with hypertension and COPD, and Group III was made up of 23 patients with COPD. The average age of patients in groups - presented as mean [standard deviation (SD)] - was as follows - Group I: 55.7 (11.2) years, Group II: 59.3 (9.2) years, and Group III: 57.8 (9.1) years. For statistical data processing, the program Statistics 10 was used.

Results

The level of blood creatinine - presented as median (Me) and interquartile range (IQR) - was statistically significantly different between the groups, and the values in the three groups were as follows - Group I (patients with hypertension): 88.3 (84.2; 102.7) μmol/l, Group II (patients with comorbid pathology of arterial hypertension and COPD): 99.0 (80.0; 115.0) μmol/l, and Group III (patients with COPD): 84.6 (75.0; 94.2) μmol/l (p=0.008). The highest level was determined in patients with hypertension and comorbid COPD, while the lowest was in the comparison group, in patients with COPD. Urinary creatinine levels were as follows - Group I: 1081.0 (578.0; 1749.0) mg/l, Group II: 1318.5 (1124.0; 1817.0) mg/l, and Group III: 822.0 (625.0; 1320.5) mg/l (p=0.08). Blood urea values were as follows - Group I: 5.7 (5.2; 6.0) mmol/l, Group II: 5.7 (4.9; 6.6) mmol/l, Group III: 5.9 (4.4; 7.7) mmol/l. The calculation of GFR revealed a statistically significant difference between the three groups - Group I: 70.5 (56.0; 83.0) ml/min, Group II: 66.5 (57.0; 77.0) ml/min, and Group III: 81.5 (70.0; 88.0) ml/min (p=0.02). The cystatin C level was 1.16 (1.03; 1.27) mg/l in Group I, 1.3 (1.22; 1.38) mg/l in Group II, and 1.05 (0.96; 1.05) mg/l in Group III.

Conclusions

In patients of all three groups, there was a decrease in renal filtration function based on the results of creatinine and cystatin C levels. Even in the group of patients with COPD without kidney disease, a decrease in GFR was observed. We noted a negative aggravating effect of COPD on renal function in patients with hypertension, which can be attributed to increased endothelial dysfunction and increased general inflammation in this group of patients.

## Introduction

The concept of comorbidity implies the formation of relationships and interactions between existing diseases, as well as the presence of common pathogenetic mechanisms, namely chronic inflammation, oxidative stress, and endothelial dysfunction. Hypertension is the most common comorbidity in patients with chronic obstructive pulmonary disease (COPD) [1].

Chronic kidney disease (CKD) and hypertension are closely linked in terms of cause and effect [2]. Decreased renal function is usually associated with increased blood pressure, and a steady increase in blood pressure accelerates the decline in renal function [2,3]. On the other hand, COPD is an inflammatory disease with systemic manifestations, and airway obstruction is not completely reversible. Patients with COPD have a higher risk of comorbidities, including lung cancer, pulmonary tuberculosis, dementia, and coronary heart disease [3,4]. These comorbidities may contribute to the progression of systemic inflammation in patients with COPD. Research and clinical observations in the last few decades have put forward the concept of comorbidity, and it can even be stated that we now live in an era of comorbidity. The probability of developing comorbidities increases with increasing life expectancy, which can be explained by both advancing age and the negative impact of the environment and living conditions [4,5].

The authors believe that patients with COPD and CKD share common risk factors, including diabetes mellitus and hypertension [1,5]. Likewise, the authors argue that patients with COPD are prone to atherosclerotic vascular damage due to general inflammatory reactions, which can also affect the renal vascular network, causing disease of small or large vessels and leading to the development of CKD [6,7,8,9]. The overall prevalence of CKD in the study population was 7.1% [2]. This result is consistent with data from studies of other populations with COPD, in which the prevalence of CKD is 4-8% [1,2].

Cystatin C is a 13 kDa protein that is considered to be one of the most important extracellular inhibitors of cysteine ​​proteases. Cystatin C is freely filtered by the glomerulus, reabsorbed and catabolized [10], but not secreted by the tubules. Over the last decade, serum cystatin C has been shown to be a sensitive serum marker of glomerular filtration rate (GFR) [10,11] and a stronger predictor of the risk of death and cardiovascular events in elderly patients than serum creatinine [11,12]. It has been established that the level of cystatin C in the urine is elevated in persons with known tubular dysfunction [13,14,15,16]. Its level does not depend on gender, age, and muscle mass and is considered to be an earlier marker of renal dysfunction than creatinine [16,17].

In this study, we aimed to examine renal function, in particular the level of cystatin C, as an early predictor of kidney damage in patients with isolated arterial hypertension, those with comorbid pathology of hypertension and COPD, and those with isolated COPD.

The abstract of this study was partially published earlier: https://lung-health.org/2022/wp-content/uploads/2022/01/ABSTRACT-BOOK_draft_2022.01.13.pdf.

## Materials and methods

The study included a total of 88 patients, who were divided into three groups: Group I consisted of 38 patients with isolated arterial hypertension, Group II comprised 27 patients with hypertension and COPD, and Group III was made up of 23 patients with isolated COPD. All patients included underwent inpatient or outpatient treatment at the Dnipro City Hospital № 4. The research design and protocol were approved by the Biomedical Ethics Commission of the Dnipro State Medical University (Protocol No. 3, November 2, 2021).

The final sample was selected after a detailed study of the data related to medical records, complaints, and general clinical examination based on the inclusion and exclusion criteria (Table 1) [4,1].

**Table 1:** Inclusion and exclusion criteria ESH/ESC: European Society of Hypertension/European Society of Cardiology; GOLD: Global Initiative for Chronic Obstructive Lung Disease; GFR: glomerular filtration rate

No.	Inclusion criteria	Exclusion criteria
1	Informed consent of the patient for processing personal data	Age of patients: ≥80 years
2	Presence of hypertension stage II, 1-3 degree (according to the recommendations of ESH/ESC (2018) for the treatment of hypertension) [4]	Presence of myocardial infarction and an acute history of cerebral circulatory disorders
3	Presence of chronic obstructive pulmonary disease A - C clinical groups, stable phase (according to recommendations of GOLD 2020) [1]	Presence of congenital and acquired kidney diseases (polycystosis, developmental abnormalities, glomerulonephritis, pyelonephritis)
4	Age of patients: ≥40 years	Presence of diabetes mellitus
5		Presence or history of cancer
6		Presence or history of arrhythmias and conduction of the heart that require medical correction
7		GFR of patients: <30 ml/min

All groups were statistically comparable in age (p≥0.05). The average age of patients in groups - presented as mean [standard deviation (SD)] - was as follows - Group I: 55.7 (11.2) years, Group II: 59.3 (9.2) years, and Group III: 57.8 (9.1) years. The duration of hypertension in Group I was 14.3 (9.8) years; in Group II, it was 14.2 (6.6) years. This difference was not statistically significant (p>0.05). Also, there were also no statistical differences in the duration of COPD between the two groups: the duration of COPD in Group II was 15.6 (7.8) years, and that in Group III was 17.5 (8.3) years (p≥0.05). Additionally, as for the severity of patients with arterial hypertension in the groups and the severity of COPD, there were no statistically significant differences. Statistical comparability of groups in terms of age, gender, the number of patients with one and two pathologies, as well as the severity of arterial hypertension and COPD, made it possible to carry out further stages of the study and process the results.

In accordance with the purpose of the study, in all patients, along with a general clinical study, a detailed study of kidney function was performed based on laboratory and instrumental methods, namely, by determining the parameters of renal function in blood and urine, and by performing an ultrasound examination of the kidneys.

Processing of the obtained research results using biostatistical methods was carried out using the STATISTICA v.6.1 software (Statsoft Inc., Tulsa, OK) (license number: AGAR909E415822FA) and included descriptive and analytical statistical methods. The test of the hypothesis about the normality of the distribution was carried out according to the Kolmogorov-Smirnov test with Lilliefors and Shapiro-Wilk corrections. Depending on the nature of the distribution, parametric and non-parametric methods were used to determine the number of observations (n). With a normal distribution of qualitative and quantitative characteristics, the average values ​​were given in the form of the arithmetic mean (M) and the mean squared SD - M (SD); in the case of a distribution of quantitative characteristics other than normal, the average values ​​were given in the form of the Me and IQR (first and third quartiles Q1-Q3 or 25%-75% percentiles): Me (25%; 75%).

## Results

According to the results of laboratory blood tests, it was found that the level of blood creatinine was statistically significantly different between the groups, and the values in the three groups were as follows - Group I (patients with isolated hypertension): 88.3 (84.2; 102.7) μmol/l, Group II (patients with comorbid pathology of arterial hypertension and COPD): 99.0 (80.0; 115.0) μmol/l, and Group III (patients with isolated COPD): 84.6 (75.0; 94.2) μmol/l (p=0.008). The highest level was determined in patients with hypertension and comorbid COPD, while the lowest was in the comparison group, in patients with COPD. A pairwise comparison revealed a statistically significant difference between the groups of patients with hypertension (Group I), hypertension and COPD (Group II), and the group of patients with COPD (Group III) (Table 2).

**Table 2:** Laboratory indicators of renal function in patients Me (25%;75%): median (interquartile range - first and third quartiles Q1-Q3 or 25%-75% percentiles) COPD: chronic obstructive pulmonary disease; GFR: glomerular filtration rate

Indicator	Patients with hypertension (n=38)	Patients with hypertension and COPD (n=27)	Patients with COPD (n=23)	P-value
Blood creatinine, μmol/l, Ме (25%; 75%)	88.3 (84.2; 102.7)	99.0 (80.0; 115.0)	84.6 (75.0; 94.2)	0.008
Urine creatinine, mg/l, Ме (25%; 75%)	1081.0 (578.0; 1749.0)	1318.5 (1124.0; 1817.0)	822.0 (625.0; 1320.5)	0.08
Urea level, mmol/l, Ме (25%; 75%)	5.7 (5.2; 6.0)	5.7 (4.9; 6.6)	5.9 (4.4; 7.7)	0.1
Urine albumin, mg/l, Ме (25%; 75%)	7.6 (4.0; 15.9)	10.6 (4.3; 24.6)	3.9 (2.0; 5.8)	0.01
GFR, ml/min, Ме (25%; 75%)	70.5 (56.0; 83.0)	66.5 (57.0; 77.0)	81.5 (70.0; 88.0)	0.02

Also, there was a trend of higher levels of urinary creatinine in patients with hypertension, especially in patients with hypertension and COPD. Urinary creatinine levels were as follows - Group I: 1081.0 (578.0; 1749.0) mg/l, Group II: 1318.5 (1124.0; 1817.0) mg/l, and Group III: 822.0 (625.0; 1320.5) mg/l (p=0.08). Blood urea values were as follows - Group I: 5.7 (5.2; 6.0) mmol/l, Group II: 5.7 (4.9; 6.6) mmol/l, and Group III: 5.9 (4.4; 7.7) mmol/l, with no pattern of higher levels of urea observed in patients depending on the presence of comorbid pathology (p=0.1).

A significant difference was found between the levels of urinary albumin, and the values were as follows - Group I: 7.6 (4.0; 15.9) mg/l, Group II:* *10.6 (4.3; 24.6) mg/l, and Group III: 3.9 (2.0; 5.8) mg/l (p=0.01). Pairwise analysis revealed a statistical difference between urinary albumin levels in patients with hypertension (Group I), hypertension and COPD (Group II), and patients with COPD (Group III). The highest urinary albumin excretion was found in patients with hypertension, especially in patients with hypertension and COPD. The calculation of GFR revealed a statistically significant difference between the three groups - Group I: 70.5 (56.0; 83.0) ml/min, Group II: 66.5 (57.0; 77.0) ml/min, and Group III: 81.5 (70.0; 88.0) ml/min (p=0.02), which indicates a statistically significant trend of decrease in the filtration function of the kidneys in patients with hypertension (Table 2).

**Figure 1:**
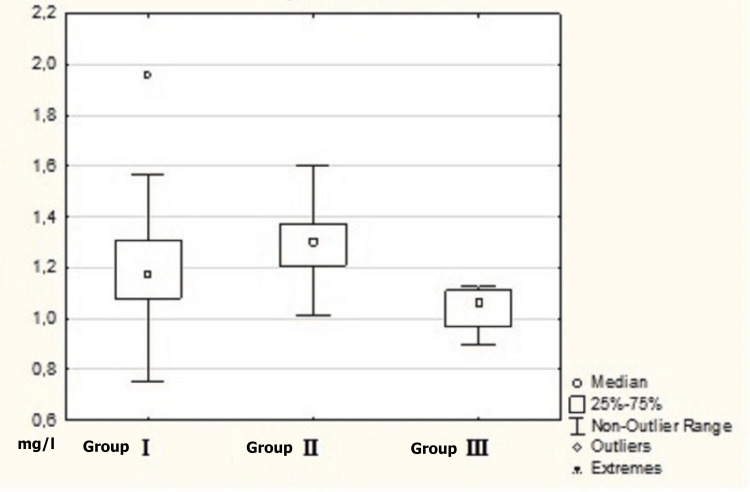
Cystatin C level in patients Group I: patients with arterial hypertension; Group II: patients with a combination of arterial hypertension and COPD; Group III: patients with COPD COPD: chronic obstructive pulmonary disease

A pairwise comparison revealed a statistically significant difference between cystatin C levels in patients with hypertension (Group I) and those with COPD (Group III): 1.16 (1.03; 1.27) mg/l and 1.05 (0.96; 1.05) mg/l respectively (p=0.02), and between patients with comorbid pathology of hypertension and COPD (Group II) and those with COPD (Group III): 1.3 (1.22; 1.38) mg/l and 1.05 (0.96;1.05) mg/l (p=0.006) respectively (Table 3, Figure 1).

**Table 3:** Cystatin C levels in patients in three groups Me (25%;75%): interquartile range (first and third quartiles Q1-Q3 or 25%-75% percentiles) COPD: chronic obstructive pulmonary disease

Indicator	Patients with hypertension (n=38)	Patients with hypertension and COPD (n=27)	Patients with COPD (n=23)	P-value
Cystatin C, mg/l, Ме (25%; 75%)	1.16 (1.03; 1.27)	1.3 (1.22; 1.38)	1.05 (0.96; 1.05)	0.04

## Discussion

Several studies have been conducted on the prevalence of CKD in patients with COPD among different populations [3,4,5]. Most of these have been single-center studies with a small sample size. However, a recent meta-analysis by Gaddam et al. showed an increased prevalence of CKD in patients with COPD even after adjusting for concomitant parameters including age, sex, BMI, and smoking status, thereby suggesting an independent association of CKD with COPD [13]. The overall prevalence of CKD in the study population was 7.1%. This result is consistent with data from studies of other populations with COPD, in which the prevalence of CKD is 4-8% [13,14,15]. Systemic inflammation can be one of the connecting links between these two conditions [16]. Several studies have identified COPD as part of a systemic inflammatory syndrome [9-12] and reported on the association between comorbidities like lung cancer, osteoporosis, progression of atherosclerosis, and CKD [13].

While the pathogenesis of CKD in patients with hypertension is known, the topic of CKD in patients with COPD is very interesting, since there are many theories on the pathogenesis of kidney damage in this group of patients. Many studies have reported a high prevalence of CKD in patients with COPD in various populations [7,9,13]. In our study, we also found a decreased kidney function in patients with COPD who did not have kidney disease or diabetes, which could have contributed to these findings. This suggests that there is a mechanism behind the development of CKD in patients with COPD.

The mechanism by which COPD potentiates the development of CKD remains unclear. However, several hypotheses have been put forward. COPD has been associated with systemic inflammation. Pro-inflammatory cytokines, especially tumor necrosis factor-alpha (TNF-α), play an important role in inflammation [13] and have been shown to increase endothelial inflammation and atherosclerosis. This inflammation is also potentially associated with the development of diabetes, muscle wasting, and kidney disease [5,9,13].

COPD is also associated with microalbuminuria, and in patients with hypoxemia and hypercapnia, effective renal blood flow is reduced. These changes may reflect the increased activity of the renin-angiotensin system observed in patients with COPD [13]. Drug treatment of COPD, such as the use of antibiotics, may contribute to the development of CKD [13].

The level of cystatin C in blood serum was determined for all patients. Cystatin C is a 13 kDa protein that is considered one of the most important extracellular inhibitors of cysteine ​​proteases. Cystatin C is freely filtered by the glomerulus, reabsorbed and catabolized, but not secreted by tubules [18]. Over the past decade, studies of serum cystatin C have shown that it is a sensitive serum marker of GFR and a stronger predictor of the risk of death and cardiovascular events in older patients than serum creatinine [19,20]. In our patients, the levels of cystatin C almost did not exceed the reference for this indicator, but the results obtained showed a statistically significant difference between its levels in patients of three groups.

Due to certain limitations of the study, such as the outbreak of the coronavirus disease 2019 (COVID-19) pandemic, our sample size was relatively small; however, we still managed to obtain results that align with those of several studies in the literature. We believe that the relatively larger sample size and the comparison of our results with the data in the literature are of scientific interest.

## Conclusions

In patients of all three groups, there was a decrease in renal filtration function based on the results of creatinine and cystatin C levels. Even in the group of patients with COPD without kidney disease, there was a decrease in GFR, but it was significantly less than that in patients with hypertension and those with comorbidity of hypertension and COPD. There was a negative effect of COPD on renal function in patients with hypertension, which can be explained by increased endothelial dysfunction and increased general inflammation in this group of patients.
